# Tumor budding may be a promising prognostic indicator in intrahepatic cholangiocarcinoma: A multicenter retrospective study

**DOI:** 10.1002/ags3.12605

**Published:** 2022-07-30

**Authors:** Hisashi Kosaka, Mitsuaki Ishida, Masaki Ueno, Koji Komeda, Daisuke Hokutou, Hiroya Iida, Fumitoshi Hirokawa, Kosuke Matsui, Mitsugu Sekimoto, Masaki Kaibori

**Affiliations:** ^1^ Department of Surgery Kansai Medical University Hirakata Japan; ^2^ Department of Pathology Osaka Medical and Pharmaceutical University Takatsuki Japan; ^3^ Second Department of Surgery Wakayama Medical University Wakayama Japan; ^4^ Department of General and Gastroenterological Surgery Osaka Medical College Takatsuki Japan; ^5^ Department of Surgery Nara Medical University Kashihara Japan; ^6^ Department of Surgery Shiga University of Medical Science Otsu Japan

**Keywords:** cancer staging, intrahepatic bile duct, intrahepatic cholangiocarcinoma, TNM classification, tumor staging

## Abstract

**Purpose:**

This retrospective study evaluated our hypothesis that high tumor budding (≥10 buds) may help determine the appropriate T category for more accurate staging of intrahepatic cholangiocarcinoma (ICC).

**Methods:**

We analyzed the clinical and histopathologic data of 235 consecutive patients with histologically confirmed ICC following hepatectomy at five university hospitals in the Kansai region of Japan between January 2009 and December 2020. ICC staging was based on the Liver Cancer Study Group of Japan (LCSGJ) staging system, 6th edition.

**Results:**

Patients with ICC with high budding showed significantly shorter disease‐specific survival (DSS) and disease‐free survival (DFS) than patients with low/intermediate budding. Cox proportional hazards regression analysis showed a hazard ratio of 2.2‐2.3 (*P* < 0.05) for high budding. Based on these results, we modified the T category of ICC in the LCSGJ staging system by adding severity of tumor budding as a fourth determinant. This proposed staging system for ICC has significantly improved the prognostic accuracy for both DSS and DFS (both: *P* < 0.05).

**Conclusions:**

High tumor budding is a new candidate for an additional determinant of the T category in staging ICC. An LCSGJ staging system containing an additional evaluation of tumor budding may lead to improved staging accuracy.

## Graphical abstract

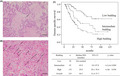

## 1 INTRODUCTION

Intrahepatic cholangiocarcinoma (ICC) is the second most common primary liver cancer, and its incidence has increased over the past three decades.^1^, ^2^ ICC is an aggressive neoplasm, and surgical treatment is considered to be the only potentially curative treatment; however, its prognosis is generally dismal, especially for patients with regional lymph node metastases or positive surgical margins.^3^, ^4^ In addition, aspects of therapy such as resection range, extent of lymphadenectomy, and type of adjuvant chemotherapy remain controversial.^5^, ^6^, ^7^, ^8^, ^9^ Therefore, the creation of a reliable staging system that accurately predicts the outcome of patients with ICC is crucial for developing a treatment strategy and assessing disease outcomes.^10^

Cancer staging is stratified according to the TNM scoring system, which includes the size and extent of the primary tumor (T), number of involved regional lymph nodes (N), and presence or absence of distant metastases (M).^11^ The TNM factors have been continuously improved with the discovery of new biologic and histologic characteristics. The Liver Cancer Study Group of Japan (LCSGJ) staging system for ICC was revised in 2019 based on the results of a nationwide survey by the LCSGJ and a study by the Japanese Society of Hepato‐Biliary‐Pancreatic Surgery (JSHBPS).^12^, ^13^ That 6th edition of the LCSGJ staging system excluded serosal invasion as a determinant of the T category, whereas a cutoff size of 2 cm and major biliary invasion were added as new determinants of the T category. In contrast, the determinants of the T category in the 2018 8th edition of the American Joint Committee on Cancer (AJCC) staging system^14^ included tumor number, tumor size, vascular invasion, visceral peritoneum invasion, and involvement of local extrahepatic tissue; and periductal invasion was excluded.^10^ Validation of the 6th edition of the LCSGJ with regard to the staging system for ICC has not yet been performed, and several validation studies of the 8th edition of the AJCC staging system have not confirmed a marked improvement in the overall prognostic performance over that of the 7th edition.^15^, ^16^, ^17^ Thus, improvements in the staging of ICC are still needed.

Tumor budding, which is a morphologic characteristic of carcinoma, is a well‐established independent prognostic factor in colorectal cancer.^18^ Tumor budding was also reported as a prognostic factor in patients with ICC and other carcinomas.^19^, ^20^, ^21^ We hypothesized that adding the determinant of high tumor budding (≥10 buds) may lead to more accurate estimation of the T category and the subsequent staging of ICC. The aims of this retrospective study were to assess the prognostic significance of high tumor budding in patients with ICC and to propose an improved postoperatively assessed T staging system that contains high tumor budding as a new determinant of the T category.

## 2 MATERIALS AND METHODS

### 2.1 Patients

We retrospectively analyzed the clinical and histopathologic data of 235 consecutive patients with histologically confirmed ICC following hepatectomy at five university hospitals in the Kansai region of Japan between January 2009 and December 2020. Clinical data were collected from each hospital, and then compiled and analyzed at Kansai Medical University.

### 2.2 Histopathologic assessment

**FIGURE 1:**
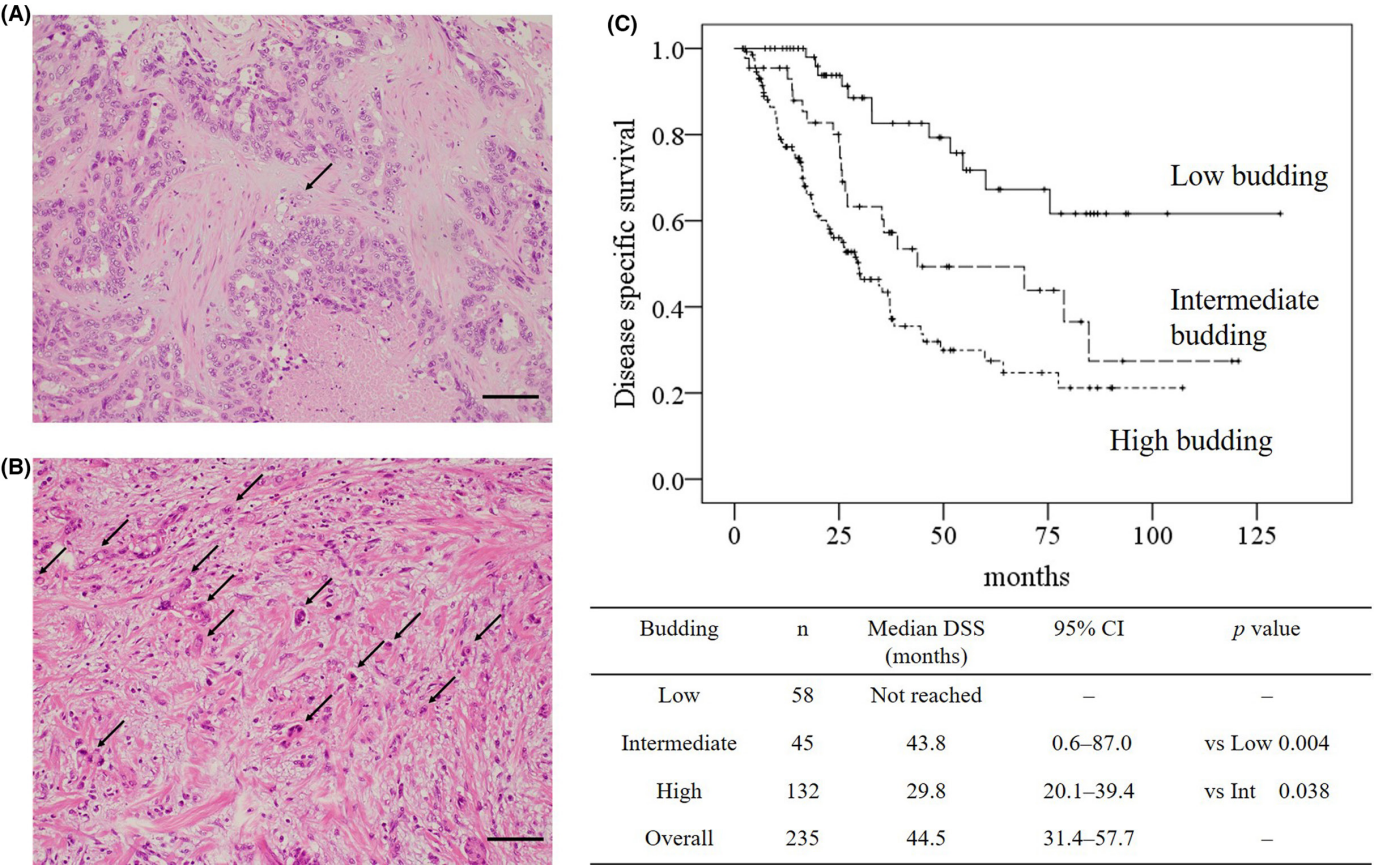
Comparison of survival based on the severity of tumor budding in intrahepatic cholangiocarcinoma. Typical images of low budding (A) and high budding (B) are shown (hematoxylin and eosin staining, objective magnification ×20). Black arrow indicates tumor buds. Black bar indicates 100 μm. The log‐rank test was used to estimate the differences between the disease‐specific survival of patients with high, intermediate, or low budding (C). DSS disease‐specific survival, CI Confidence interval

Tumors were excised from patients at each of the five university hospitals, fixed in 10% formalin, and embedded in paraffin. Serial sections of each tumor were stained with hematoxylin and eosin. The stained sections were collected and analyzed at Kansai Medical University. Tumor budding was blindly evaluated by two pathologists in accordance with the 2016 International Tumor Budding Consensus Conference (ITBCC). Tumor buds were considered to be a single tumor cell or a cluster of up to four tumor cells on hematoxylin and eosin staining in one hotspot at the invasive front, in a field measuring 0.785mm^2^.^18^ Ten separate fields (20x objective) along the invasive front are scanned prior to counting of tumor buds in the single selected hotspot to ensure that the field with the highest tumor budding is selected.^18^ Tumor budding was categorized by a three‐tier system in accordance with ITBCC recommendation as follows: low (zero to four buds), intermediate (five to nine buds), or high (≥10 buds). Typical images of tumor budding are shown in Figure 1.

### 2.3 Creation of the modified T category in the staging system for ICC

ICC staging was evaluated in accordance with the 6th edition of the LCSGJ staging system and 8th edition of the AJCC staging system.^13^, ^14^ In this study, we uniquely proposed staging system based on the 6th edition of the LCSGJ staging system. The original 6th edition consisted of the following three determinants of the T category as follows: number of tumors, sizes of tumors, and severity of vascular or major biliary invasion. In contrast, our proposed criteria have added the severity of tumor budding as the fourth determinant of the T category. The details on the original 6th edition of the LCSGJ staging system and proposed staging system for ICC are shown in Table 1. The 6th edition of the LCSGJ staging system is originally adapted for patients with mass forming type, whereas 8th edition of the AJCC staging system is used for all patients with ICC regardless of tumor type such as mass forming, periductal infiltrating, and intraductal growth type. In this study, study cohort includes all tumor types to compare various staging systems.

**TABLE 1:** Comparisons between criteria of the original 6th edition of the LCSGJ staging system and the proposed staging system

6th edition of the LCSGJ	Proposed staging system
Determinants of the T category Solitary tumor Tumor size ≤2 cm No vascular or major biliary invasion (Vp0, Va0, B0‐B2)	Proposed determinants of the T category Multiple tumors Tumor size >2 cm Vascular or major biliary invasion (Vp≥1, Va≥1, B3‐B4) High budding (>10 buds)
T category classification	Proposed T category classification
T1: All determinants are fulfilled	T1: None of the determinants is fulfilled
T2: Only two determinants are fulfilled	T2: One of the determinants is fulfilled
T3: Only one determinant is fulfilled	T3: Two of the determinants are fulfilled
T4: None of the determinants is fulfilled	T4: Three of the determinants are fulfilled T5: All determinants are fulfilled
Stage classification	Proposed stage classification
I: T1N0M0	I: T1N0M0
II: T2N0M0	II: T2N0M0
III: T3N0M0	III: T3N0M0
IVA: T4N0M0 or T1‐T3N1M0	IVA: T4N0M0 or T1‐T3N1M0
IVB:T4N1M0 or AnyTAnyNM1	IVB:T4N1M0 or AnyTAnyNM1 T5AnyNAnyM

*Note*: LCSGJ Liver Cancer Study Group of Japan, T Tumor classification, N Lymph node status, M Metastasis status, Vp0 No portal vein invasion, Vp≥1 Invasion to portal vein and/or its branch, Va0 no arterial invasion, Va≥1 Invasion to hepatic artery and/or its branch, B0‐B2 No biliary invasion or minor biliary invasion within the second branch of the bile duct, B3‐B4 biliary invasion to the first branch of the bile duct and/or common bile duct.

### 2.4 Statistical analysis

Data are expressed as numbers with percentages or medians with interquartile ranges (IQRs). The Shapiro‐Wilk test was used to assess the normality of continuous variables. The Student t test or Welch test were performed after the Levene test for normally distributed data, and the Mann‐Whitney U test was performed for non‐normally distributed data. The Fisher exact test was used for nominal scale data. Comparisons were considered statistically significant at *P* < 0.05. The Kaplan‐Meier method and log‐rank test were performed to assess differences between disease‐specific survival (DSS) and disease‐free survival (DFS). DSS begins at the time of surgery and ends at the time of disease‐specific death. Patients who died from other obvious reason such as heart attack, cerebral hemorrhage were not counted as disease‐specific death. DFS was determined from an analysis of 231 study patients who had R0 or R1 resection margins. The four remaining study patients had R2 resection margins and were excluded from the analysis. Cox proportional hazards regression analysis was performed by a forward stepwise method to detect independent risk factors of DSS and DFS. Hazard ratios with 95% confidence intervals (CIs) were estimated. All statistical analyses were performed with the IBM SPSS ver. 22 software package for Windows (IBM Japan Ltd., Tokyo, Japan).

### 2.5 Ethics

This study was approved by the institutional review board of Kansai Medical University (Approval number: 2019322). It was performed in accordance with the Declaration of Helsinki.

## 3 RESULTS

### 3.1 Background characteristics

The background characteristics of an entire cohort are shown in Table 2. The median age of the patients was 71 years, and 69% were male. Fifty‐one patients have hepatic virus infection (21.7%). Liver dysfunction was not notable in median values of ALBI score (−2.82) and ICG (10.2%). Median value of FIB4 index indicated intermediate range of liver fibrosis (2.05). Median value of CA19‐9 was slightly higher than standard value (44.0 U/mL), whereas CEA was within normal limit (2.8 ng/mL). A rate of preoperative accurate diagnosis as ICC was 80.9%. Majority of surgical procedure was bisectionectomy (56.2%) and regional lymphadenectomy was undertaken in 48.9%. Adjuvant chemotherapy was administered to 51% of the study patients. Of the 235 consecutive patients with tumors histologically confirmed to be ICC, 132 (56%) had high‐budding tumors and 103 (44%) had low‐/intermediate‐budding tumors.

**TABLE 2:** Background characteristics

Variable	N (%) or median (IQR)
N	235
Age, years	71.0 (66.0‐76.0)
Gender, Male	162 (68.9)
Hepatitis, HBV/HCV/both	34/20/3 (14.5/8.5/1.3)
ALBI score	−2.82 (−3.04 ‐ −2.48)
FIB4 index	2.05 (1.45‐2.85)
ICG (%)	10.2 (7.3‐14.1)
CEA (ng/mL)	2.8 (1.9‐4.7)
CA 19‐9 (U/mL)	44.0 (13.0‐225.0)
Preoperative diagnosis as ICC	190 (80.9)
Hepatectomy procedure	
Less than sectionectomy	54 (23.0)
Sectionectomy	32 (13.6)
Bisectionectomy	132 (56.2)
Trisectionectomy	17 (7.2)
Extrahepatic bile duct resection	45 (19.1)
Regional lymphadenectomy	115 (48.9)
Laparoscopic approach	57 (24.3)
Operative time (min)	370.0 (274.0‐492.0)
Blood loss (mL)	540.0 (175.0‐1040.0)
Clavien‐Dindo score ≥ IIIa and more	66 (28.1)
Mortality within 90 days after surgery	6 (2.6)
Adjuvant chemotherapy	120 (51.1)
Tumor budding, Low/Intermediate/High	58/45/132 (24.7/19.1/56.2)

*Abbreviations*: ALBI score, Albumin‐bilirubin score; CA19‐9, Carbohydrate antigen 19‐9; CEA, Carcinoembryonic antigen; FIB4 index, Fibrosis‐4 index; HCV, Hepatitis C virus; ICC, Intrahepatic cholangiocarcinoma; ICG, Indocyanine green; IRQ, Interquartile range; HBV, Hepatitis B virus.

### 3.2 Significant effects of tumor budding on patient survival

Tumor budding was evaluated in accordance with the 2016 ITBCC. The median number of tumor buds were 1.5 (IQRs: 0.0‐3.0) in low budding, 7.0 (IQRs: 6.0‐8.0) in intermediate, 15.5 (IQRs: 12.0‐23.0) in high budding, and 11.0 (IQRs: 6.0‐17.0) in overall. The concordance rate of three‐tiers between two pathologists was 99.6% and the kappa coefficient was 0.993 (*P* < 0.001). The results of Kaplan‐Meier analysis and log‐rank tests of the DSS of patients stratified by the severity of tumor budding are shown in Figure 1. The median DSS of patients with high‐budding tumors was 14 months shorter than the median DSS of patients with intermediate‐budding tumors (29.8 months vs 43.8 months, respectively; *P* < 0.05). The median DFS of patients with high‐budding tumors was 17 months shorter than the median DFS of patients with intermediate‐budding tumors (10.2 months vs 27.0 months, respectively; *P* < 0.05).

Table 3 shows the results of a Cox proportional hazards regression analysis that investigated the effect of high‐budding tumors on DSS and DFS by comparing with perioperative findings and determinants of the 6th edition of the LCSGJ stage. High tumor budding had the second‐highest hazard ratios (HRs) for DSS and DFS after vascular or major biliary invasion (DSS: vascular or major biliary invasion: HR = 3.4 high tumor budding: HR = 2.2, *P* < 0.05; DFS: vascular or major biliary invasion: HR = 2.5, high tumor budding: HR = 2.3, *P* < 0.05). High tumor budding demonstrated significant impact on survival, whereas the cohort of high and intermediate tumor budding could not.

**TABLE 3:** COX proportional hazards regression analysis of the prognostic ability of perioperative findings

Variable	Disease‐specific survival		Disease‐free survival	
	Hazard ratio (95% CI)	*P* value	Hazard ratio (95% CI)	*P* value
Age, years	–	0.861	–	0.393
Gender, male	–	0.677	–	0.385
Hepatitis, HBV / HCV / both	1.4 (1.0‐1.8)	0.033	–	0.076
ALBI score	1.6 (1.1‐2.5)	0.017	–	0.140
FIB4 index	–	0.159	–	0.623
ICG, %	–	0.353	–	0.594
CEA, ng/mL	–	0.220	–	0.620
CA 19‐9, U/mL	1.0 (1.0‐1.0)	0.003	–	0.059
Tumor size >2 cm	2.2 (1.1‐4.5)	0.032	–	0.099
Vascular or major biliary invasion	3.4 (1.9‐6.1)	<0.001	2.5 (1.5‐4.1)	<0.001
Multiple tumors	1.6 (1.0‐2.6)	0.044	2.0 (1.3‐3.2)	0.001
Lymph node metastasis	2.1 (1.3‐3.5)	0.003	2.0 (1.3‐3.1)	0.002
Resection margin, positive	–	0.795	–	0.452
Budding, high	2.2 (1.3‐3.5)	0.002	2.3 (1.5‐3.5)	<0.001
Budding, high and intermediate	–	0.273	–	0.145

*Abbreviations*: ALBI score, Albumin‐bilirubin score; CA19‐9, Carbohydrate antigen 19–9; CEA, Carcinoembryonic antigen; CI, Confidence interval; FIB4 index, Fibrosis‐4 index; HBV, Hepatitis B virus; HCV, Hepatitis C virus; ICG, Indocyanine green.

### 3.3 Histopathologic characteristics stratified by severity of tumor budding

Differences in tumor histopathologic characteristics between the high‐budding and the low‐/intermediate‐budding cohorts are shown in Table 4. In the entire study population, the majority of tumors were mass‐forming (79%) and moderately differentiated (55%). The intraductal growth type was rare among patients with high‐budding tumors, and significantly fewer patients with high‐budding tumors had well‐differentiated tumors (*P* < 0.05). The size of the high‐budding tumors was slightly larger than that of low‐/intermediate‐budding tumors (39.5 mm vs 35.0 mm, respectively; *P* < 0.05). Significantly higher proportions of patients with high‐budding vs low‐budding tumors had vascular or major biliary invasion (77.3% vs 58.3%, respectively; *P* < 0.05) and regional lymph node metastases (54.3% vs 16.3%, respectively; *P* < 0.05). The differences between cohorts in the proportions of patients with tumors with R2 margins were not significant (high‐budding: 2.3% vs low‐/intermediate‐budding: 1.9%, *P* > 0.05).

**TABLE 4:** Histopathologic characteristics stratified by severity of tumor budding

Variable	Overall	Tumor budding		*P* value
		Low/intermediate	High	
N	235	103	132	–
Tumor type				0.024
Mass forming	185 (78.7)	78 (75.7)	107 (81.1)	
Periductal infiltrating	36 (15.3)	14 (13.6)	22 (16.7)	
Intraductal growth	14 (6.0)	11 (10.7)	3 (2.3)	
Differentiation				<0.001
Well	65 (27.7)	44 (42.7)	21 (15.9)	
Moderate	129 (54.9)	43 (41.7)	86 (65.2)	
Poor	25 (10.6)	10 (9.7)	15 (11.4)	
Other	16 (6.8)	6 (5.8)	10 (7.6)	
Tumor size	36.0 (25.0‐58.0)	35.0 (20.0‐52.0)	39.5 (27.3‐64.5)	0.042
Multiple tumors	36 (15.3)	11 (10.7)	25 (18.9)	0.081
Vascular or major biliary invasion	162 (68.9)	60 (58.3)	102 (77.3)	0.002
Perforating the visceral peritoneum	70 (29.8)	25 (24.3)	45 (34.1)	0.102
Regional lymph node metastasis^a^	46/119 (38.7)	8/49 (16.3)	38/70 (54.3)	<0.001
Resection margin status, R0/1/2	202/29/4 (86.0/12.3/1.7)	93/9/1 (90.3/8.7/1.0)	109/20/3 (82.6/15.2/2.3)	0.234

*Note*: CBD Common bile duct.

^a^ Regional lymph node metastasis was calculated as the number of lymph node‐positive patients among all patients who underwent regional lymphadenectomy or sampling.

### 3.4 Validation of the stage of the 6th edition of the LCSGJ staging system and 8th edition of the AJCC staging system

**FIGURE 2:**
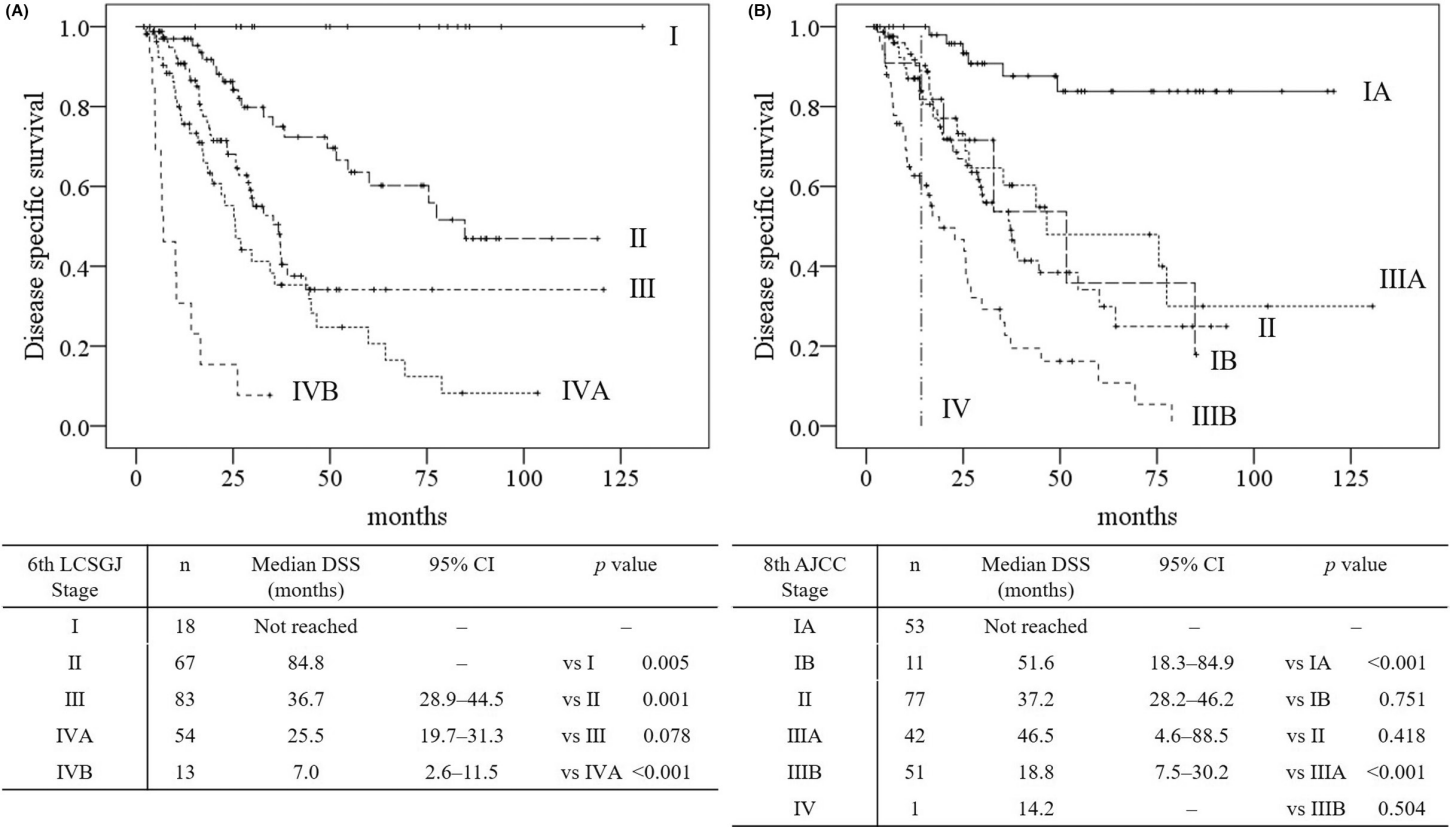
Comparison of the stratification of disease‐specific survival based on stage of disease with the 6th edition of the LCSGJ staging system, 8th edition of the AJCC staging system. Kaplan‐Meier curves demonstrate the disease‐specific survival of patients with disease stage stratified according to the 6th edition of the LCSGJ staging system (A), 8th edition of the AJCC staging system (B). LCSGJ Liver Cancer Study Group of Japan, AJCC American Joint Committee on Cancer, DSS disease‐specific survival, CI Confidence interval

Figure 2A shows the Kaplan‐Meier curves of the DSS of patients stratified based on stage of disease according to the 6th edition of the LCSGJ staging system. No differences were seen between the DSS of patients with III and IVA disease (*P* = 0.078). On the other hand, the 6th edition of the LCSGJ staging system shows all stages, with statistical differences between the DFS of the patients at every stage (all, *P* < 0.05). Figure 2B shows the Kaplan‐Meier survival curves of the DSS of patients stratified based on stage of disease according to the 8th edition of the AJCC staging system. Some overlaps were seen between the DSS of patients with IB vs II (*P* = 0.751), II vs IIIA (*P* = 0.418), and IIIB vs IV (*P* = 0.504). In addition, significant overlaps were also seen between the DFS of patients with IB vs II and II vs IIIA (all, *P* > 0.05).

### 3.5 Validation of the T category of the 6th edition of the LCSGJ staging system and 8th edition of the AJCC staging system

**FIGURE 3:**
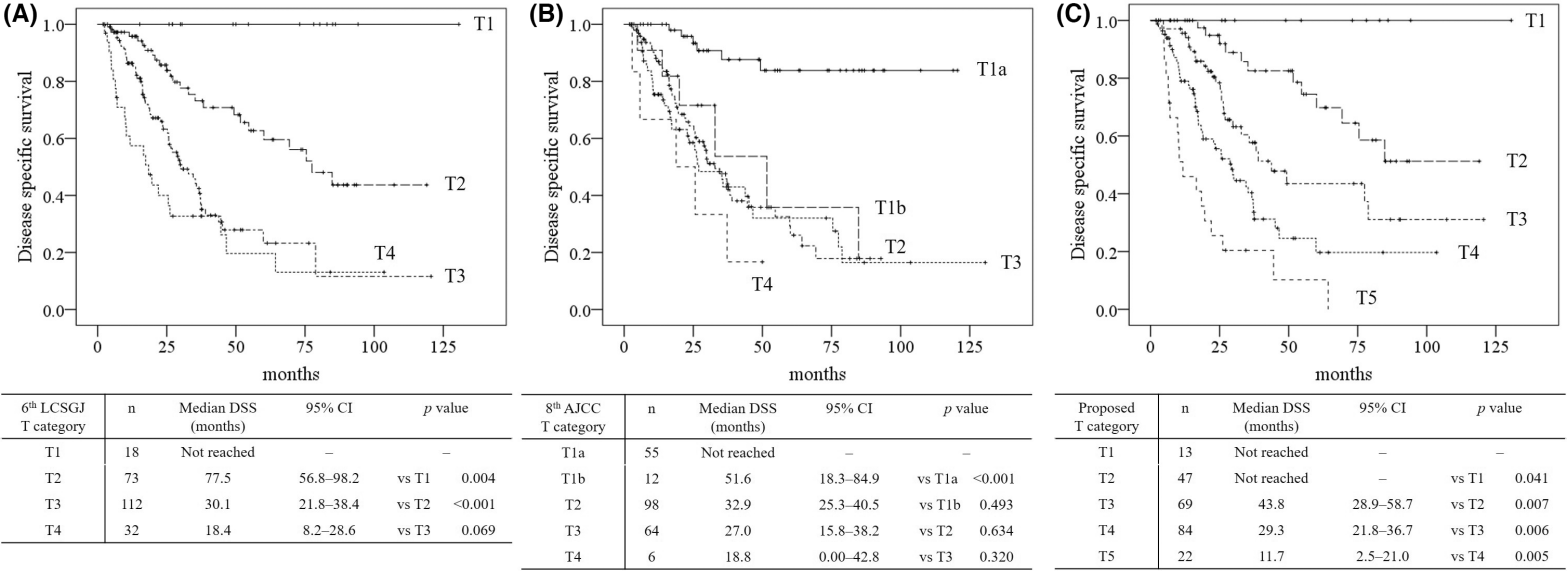
Comparison of the disease‐specific survival of patients with T categories stratified according to the 6th edition of the LCSGJ staging system, 8th edition of the AJCC staging system and the proposed staging system. Kaplan‐Meier curves show the disease‐specific survival of patients with T categories stratified according to the 6th edition of the LCSGJ staging system (A), 8th edition of the AJCC staging system (B), and the proposed staging system (C). LCSGJ Liver Cancer Study Group of Japan, AJCC American Joint Committee on Cancer, DSS disease‐specific survival, CI Confidence interval

The 6th edition of the LCSGJ stage and 8th edition of the AJCC staging system were validated by using this study cohort, as shown in Figure 3. Figure 3A shows the Kaplan‐Meier survival curves of the DSS of patients stratified by the T categories of the 6th edition of the LCSGJ. No differences were seen between the DSS of patients with T3 and T4 disease (*P* = 0.069). In addition, no differences were also seen between the DFS of patients with T3 and T4 disease (*P* = 0.068). Figure 3B shows the Kaplan‐Meier survival curves of the DSS of patients stratified by the T categories of 8th edition of the AJCC staging system. No differences were seen between the DSS of patients with T1b, T2, T3, and T4 (all, *P* > 0.05). In addition, no differences were also seen between the DFS of patients with T1b, T2, T3, and T4 (all, *P* > 0.05).

### 3.6 Improvements in the stratification of determinants with the proposed T category compared with the T category of the 6th edition of the LCSGJ

Because tumor budding was revealed to have a significant effect on survival, the severity of tumor budding was added to the three determinants of the T category of the 6th edition of the LCSGJ T category as a candidate for a fourth determinant (Table 1). When patients were restaged according to the proposed T category, the numbers of patients with T2 and T3 disease decreased (T2: original T category n = 73 → proposed T category n = 47; T3: original n = 112 → proposed n = 69). The numbers of patients with T4 disease increased with the addition of the proposed T category (T4: original n = 32 → proposed n = 84). The number of patients with newly created T5 disease was 22. Figure 3C shows the Kaplan‐Meier curves of the DSS of patients stratified by the proposed T categories. The differences between each T category were significant (all, *P* < 0.05). In addition, the differences between the DFS of patients with each T category were also significant (all, *P* < 0.05). Cox proportional hazards regression analysis that investigated the prognostic ability on DSS by comparing with the 6th edition of the LCSGJ T category, the T category of 8th edition of the AJCC staging, and the proposed T category was performed. The proposed T category demonstrated highest HRs (DSS: the proposed T category: HR = 2.0 8th AJCC T category: HR = 1.4, *P* < 0.05), whereas 6th edition of the LCSGJ T category did not demonstrate statistical significance (*P* > 0.05).

## 4 DISCUSSION

In this multicenter retrospective study of patients in the Kansai region of Japan, we validated the 6th edition of the LCSGJ staging system for ICC. In our cohort, significant overlap was seen for the DSS and DFS of patients with T3 and T4 disease. Consequently, the DSS of patients with stage III and IVA disease also overlapped significantly. One reason accounting for the overlap may be differences in the years during which the supporting studies were conducted. The 6th edition of the LCSGJ staging system for ICC was based on the results of an analysis of a nationwide survey performed between 2000 and 2005 and a project study of the JSHBPS that used a patient cohort enrolled between 1995 and 2004.^12^, ^13^ In contrast, our study assessed data from patients who underwent surgery between January 2009 and December 2020; thus, patients backgrounds were based on an improved therapeutic strategy, which may have led to inappropriate overlap.^5^, ^6^, ^9^, ^22^

The 6th edition of the LCSGJ staging system contains three determinants for the T category, including number of tumors, tumor size (cutoff: 2 cm), and existence of vascular or major biliary invasion.^13^ In this study, The HRs of these three determinants of T category for both DSS and DFS were in the range of 1.6‐3.4. On the other hand, high tumor budding demonstrated significantly high HRs for both DSS and DFS (HR: 2.2 for DSS, HR: 2.3 for DFS). The HRs of the high tumor budding were similar with HRs of original three determinants of T category. Based on these findings, high tumor budding was added as a fourth determinant of the T category in the proposed modified edition of the LCSGJ staging system. Number of tumors, tumor size (cutoff: 2 cm), existence of vascular or major biliary invasion, and high tumor budding were counted as same value in modified T category. Previous studies have reported that tumor budding (tumors with ≥5 buds) was a powerful prognostic indicator for both overall survival and recurrence free survival.^21^, ^23^ These reports also provide a strong rationale for high tumor budding as a fourth determinant of the T category.

An accurate preoperative diagnosis of ICC is sometimes difficult, especially for peripheral tumors that are found to be ICC.^5^ Only half of the patients with peripheral ICC receive a preoperative diagnosis of ICC, and regional lymph nodes have only been dissected in 25% of patients with peripheral ICC.^5^ Because hepatocellular carcinoma usually does not require lymph node dissection, lymphadenectomy was omitted in half of the patients with peripheral ICC. Thus, the majority of cases of peripheral ICC are missing information on metastatic lymph nodes. In our study, we first confirmed the prognostic significance of high tumor budding for accurate staging of ICC. High tumor budding was added as a determinant in the modified T category. Restaging demonstrated decreases in the number of patients with T2 and T3 disease (original T2 disease: n = 73 → proposed T2 disease n = 47, original T3: n = 112 → proposed T3: n = 69) whereas the numbers of patients with T4 and T5 disease increased (original T4 disease: n = 32 → proposed T4: n = 84, proposed T5: n = 22). One reason accounting for improved staging might be that tumor budding compensated for the lack of information on the severity of the disease, such as no data on metastatic regional lymph nodes. In our study, the frequency of regional lymph node metastasis was three‐fold greater in patients with high‐budding tumors than in patients with low‐ and intermediate‐budding tumors. Previous reports also suggest that tumor budding is associated with lymph node metastasis in patients with ICC or perihilar cholangiocarcinoma.^21^, ^24^ Namely, patients with metastatic lymph nodes who did not undergo lymphadenectomy might be identified as T4N0, which is the same as patients with stage IVA showing T1‐3N1 disease.

Although this was a multicenter study, the number of patients with ICC in this study is limited. A larger study that comprises an analysis of a nationwide survey of ICC or a project study from a surgical society may be needed to confirm the prognostic significance of high tumor budding for accurate staging of ICC.

## 5 CONCLUSIONS

High tumor budding is a new candidate for a determinant of the T category for the staging of ICC. The proposed staging system that is based on an additional evaluation of tumor budding may contribute to improving the accuracy of staging of ICC.

## DISCLOSURE

Funding: There was no financial support for this study

Approval of the research protocol: This study was approved by the institutional review board of Kansai Medical University (Approval number: 2019322).

Informed Consent: Informed consent was obtained from all subjects involved in the study.

Conflict of interest: The authors declare no conflict of interest.

Author contributions: HK designed and performed statistical analysis and edited the manuscript. MK and MS supervised this study. MI evaluated all histopathological data. MU, KK, DH, HI, FH, and KM collected the clinical data in each university hospital.
